# Molecular Typing of *Mycobacterium tuberculosis* Strains with a Common Two-Band IS*6110* Pattern

**DOI:** 10.3201/eid0811.020292

**Published:** 2002-11

**Authors:** Kerry H. Lok, William H. Benjamin, Michael E. Kimerling, Virginia Pruitt, Donna Mulcahy, Nancy Robinson, Nancy B. Keenan, Nancy E. Dunlap

**Affiliations:** * University of Alabama School of Medicine, Birmingham, Alabama, USA; † Alabama Department of Public Health, Montgomery, Alabama, USA

**Keywords:** tuberculosis (TB) genotyping, molecular epidemiology, homeless, variable number of tandem repeats (VNTR), spoligotype

## Abstract

We conducted a population-based molecular typing of all *Mycobacterium tuberculosis* isolates obtained in Alabama since 1994. Of 2,452 isolates, 1,013 (41%) had fewer than 6 bands of IS*6110*; 348 (14%) had a single two-band pattern (JH2). With conventional epidemiologic methods, we identified three groups of related patients with JH2 isolates. Spoligotyping and pattern of variable number of tandem repeats identified 10 molecular groups; two found by conventional methods were subdivided.

 To achieve the national goal of tuberculosis (TB) elimination, disease control efforts must be based on a thorough understanding of transmission patterns among the general population and among groups at particular risk (1,2). In close coordination with the Alabama Department of Public Health, we conducted a program of population-based molecular typing of all *Mycobacterium tuberculosis* isolates identified since 1994 in Alabama. The state contains a mixture of rural and urban settings with a stable population, and the proportion of tuberculosis cases among the foreign-born and HIV-positive groups is low (2).

## The Study

Through May 2001, we typed 2,452 isolates using the IS*6110* restriction fragment length polymorphism (RFLP) method and have combined the results of molecular analysis with an aggressive contact investigation program. We found that 1,013 (41%) of the IS*6110* patterns had ≤6 bands, including 348 (14%) with a two-band RFLP pattern called JH2. Conventional epidemiologic techniques identified three groups with related JH2. Resistance to a single drug (isoniazid or streptomycin) was associated with two groups, and homelessness was associated with a large group. Results from the latter group have been previously reported (3,4).

To further characterize JH2, the most common RFLP pattern statewide, we used two polymerase chain reaction (PCR)–based methods for secondary typing methods, spacer oligonucleotide type (spoligotyping) and variable number of tandem repeats (VNTR). Isolates were selected for further molecular typing to determine which method or combination of methods could help us understand the epidemiology of this important RFLP pattern in Alabama and the extent of molecular relatedness in the JH2 cluster.

We selected 102 (29%) of the 348 JH2 pattern isolates from the Alabama genotype database that represented two-band IS*6110* RFLP strains. Groups of isolates identified through conventional epidemiologic links were selected for evaluating the secondary molecular typing techniques. Fifteen isolates from northeast Alabama were obtained from a conventional epidemiologic investigation that showed a convenience store as the site of transmission (5). Twenty-four isolates from central Alabama came from a previous investigation (3,4) of a TB outbreak in a large community of homeless persons, and four isolates were from a known outbreak on a school bus in southeast Alabama. The remaining 59 isolates were selected from the general strain collection for comparison. The selection was based on availability of viable cultures for DNA extraction and the year that these outbreaks occurred. Two isolates from the nonclustered strain collection did not generate an adequate VNTR pattern and were excluded from the final analysis. Therefore, 100 isolates were available for the combined analysis.

Isolates of *M. tuberculosis* were cultured on Lowenstein-Jensen or 7H11 Middlebrook plates for at least 4 weeks before DNA extraction. Chloroform-isoamyl alcohol was used to extract chromosomal DNA from isolates, and IS*6110* RFLP typing was performed according to international standards (6,7). All fingerprint images were scanned into a computerized database and Whole Band Analyzer, version 4.01 (Genomic Solutions Inc., Ann Arbor, MI, USA) was used. Spoligotyping was used as a secondary typing method on the basis of the presence or absence of 43 variable spacers in the direct repeat region of *M. tuberculosis* (3,8–12). Spoligotyping membranes were used according to manufacturer’s recommendations (Isogen Bioscience BV, Bilthoven, the Netherlands), and the spacer regions were numbered as reported previously (8). A spreadsheet was used to analyze the spoligotyping results. For national database reporting purposes, we converted the spoligotyping image into an octal digital format (13).

VNTR typing was used to further investigate strains that matched by both IS*6110* and spoligotyping methods or had a known epidemiologic link. This typing yielded 86 samples. As previously described (14), we used seven sets of primers and individually amplified regions with known informative tandem repeats. Electrophoresis was conducted on the PCR products in agarose. The ethidium bromide–stained gel was imaged to determine the size of the amplicon and the number of tandem repeats in each locus. We used a DNA ladder and amplicons from strain H37Rv to determine fragment sizes, according to Frothingham and Meeker-O’Connell. (14).

## Conclusions

We used spoligotyping to separate the JH2 isolates into 20 different subtypes. Four of the 20 patterns contained >2 isolates. Eighty-four of the 100 isolates remained clustered after spoligotyping. The largest spoligotyping group (designated CDC spoligo 9) contained 55 isolates. The other 3 clustered patterns were designated CDC spoligotype 3 (n=12 isolates), CDC spoligotype 545 (n=14 isolates), and CDC spoligotype 550 (n=3 isolates). Two of the clusters previously identified through contact epidemiology were subdivided.

Eighty-six strains were typed with VNTR, which generated 15 different VNTR profiles (Table), of which the largest group contained 25 isolates. Nine profiles contained more than one isolate, and six isolates were unique. The number of isolates in the groups varied from 2 to 25. The largest cluster (homeless, n=24), which was identified previously through contact epidemiology, was subdivided into 10 subtypes.

**Table:** Comparison of a common two-band IS*6110* RFLP pattern with spoligotyping and VNTR^a^

Spoligotyping		VNTR		Related to conventional outbreak		
Octal (CDC designation)	No. of isolates	VNTR results (AL designation)	No. of isolates	Store	Bus	Homeless
777776777760601(0009)	55	6,3,2,4,2,3,2 (01)	22			2
		6,3,2,3,2,2,2 (02)	12		4	4
		6,3,2,4,2,3,3 (03)	8			2
		6,3,2,3,2,2,3 (04)	4			3
		6,3,2,2,2,2,2 (05)	3			3
		6,3,2,3,2,3,2 (06)	2			
		6,3,2,3,2,3,3 (07)	2			1
		6,3,2,4,2,2,2 (08)	1			
		6,2,2,3,2,2,2 (09)	1			1
037776777760601(0545)	14	6,3,2,3,2,4,2 (10)	14	14		
777776777760771(0003)	12	6,3,2,2,2,1,2 (11)	7			5
		6,3,2,3,2,3,2.5 (12)	1			
		6,3,2,2,2,1,3 (13)	1			
		6,3,1,2,2,2,3 (14)	1			1
		6,3,2,3,2,2,2 (02)	1			1
		6,3,2,3,2,3,3 (07)	1			
001776777760601(0550)	3	6,3,2,4,2,3,2 (01)	3			
777776374360711(1445)	1	6,3,2,1,2,3,2.5 (15)	1			1
037766677760601(n/a)	1	6,3,2,3,2,4,2 (10)	1	1		
Total	86		86	15	4	24

^a^RFLP, restriction fragment length polymorphism; VNTR, variable number of tandem repeats, CDC, Centers for Disease Control and Prevention.

 Among those isolates with both secondary typing results, we identified 10 clusters plus 9 unique isolates. Clustered cases accounted for 77 of 100 cases. The largest spoligotype cluster of 55 isolates (CDC spoligotype 9), after combination with the VNTR results, was reclassified into nine unique profiles, including seven different clusters. The largest cluster had 22 isolates. CDC spoligotype 3 was divided into a single cluster of seven isolates and five unique profiles. CDC spoligotype 545 and CDC spoligotype 550 each represented single clusters.

The two-band JH2 pattern was the most common IS*6110* fingerprint found during nearly 8 years of TB genotyping surveillance in Alabama. This pattern matches National Tuberculosis Genotyping Fingerprint Pattern (NTGFP) 00016 in the database of the National Tuberculosis Genotyping Surveillance Network. 00016 was also the most common fingerprint pattern found during 5 years of the TB genotype project and was reported from all seven sentinel surveillance sites with a final frequency of 5% of all isolates.

 Pattern JH2 represents a group of similar IS*6110* patterns. The smaller band at 1.46 kb is conserved, and the larger band is located in direct repeat 24, a common insertion site. Consequently, the size of the larger band varies, depending on the number of direct repeats located upstream of spacer 24. A strain’s spoligotype can predict the size of the larger band; the larger bands analyzed in our study were 4.5 kb–4.8 kb. The resolution of the gel and the Whole Band Analyzer (Genomic Solutions, Inc.) do not discriminate well between these sizes, which could result in different spoligotypes of a single IS*6110* insertion pattern being considered as different RFLP patterns.

 The prominence of pattern JH2 (NTGFP 00016) statewide and throughout the network suggests an older, more stable IS*6110* pattern. A group of strains likely spread throughout the general population early in the TB epidemic (19th and early 20th centuries) and remain endemic in the 21st century in the United States. Although this pattern is found rarely in European isolates, some countries in Africa report a high prevalence (15).

 Despite large numbers of the IS*6110* pattern found in Alabama, we could define only a few distinct groups through conventional epidemiologic methods. Using VNTR and spoligotyping techniques to reexamine some of these clusters, we gained a better understanding of disease transmission among community groups. Two clusters previously classified by conventional epidemiologic methods were confirmed by this study. Both clusters were associated with unique drug resistance patterns in outbreaks involving a school bus (isoniazid resistance) and a neighborhood convenience store (streptomycin resistance). The largest cluster, which involved the homeless community, revealed multiple molecular-based subclusters not identifiable by routine epidemiologic study, drug susceptibility data, or both. These subclusters signify the actual genetic diversity of this most prevalent IS*6110* pattern.

 The importance of secondary typing for low -copy IS*6110* strains is well accepted (9–12)(as is the necessity of combining conventional methods with those of molecular epidemiology (2–4). We found that VNTR is particularly well suited as a secondary typing method for the common JH2 pattern. However, the VNTR method is more expensive and time-consuming because seven PCRs are required. VTNR may be a preferred method for TB research, but not for widespread TB surveillance. Automated systems could reduce overall costs, however. While spoligotyping was less discriminatory in this study, we believe it should be evaluated carefully for use in other settings.
